# Preparation of a Supramolecular Assembly of Vitamin D in a β-Cyclodextrin Shell with Silver Nanoparticles

**DOI:** 10.3390/molecules30244823

**Published:** 2025-12-18

**Authors:** Ryszhan Y. Bakirova, Serik D. Fazylov, Ainara S. Iskineyeva, Akmaral Zh. Sarsenbekova, Aleksandr K. Sviderskiy, Olzhas T. Seilkhanov, Ayaulym K. Mustafayeva, Anel Z. Mendibayeva, Bolatkul Dh. Ashirbekova, Mereke T. Agedilova, Gaukhar Khabdolda

**Affiliations:** 1 Department of Internal Diseases, Karaganda Medical University, Karaganda 100012, Kazakhstan; bakir15@mail.ru (R.Y.B.);; 2 Institute of Organic Synthesis and Coal Chemistry, Karaganda 100008, Kazakhstan; anenyawa@mail.ru; 3 Department of Technology of Food and Processing Industries, Saken Seifullin Kazakh Agrotechnical University, Astana 010000, Kazakhstan; iskeneeva_aynara@mail.ru; 4 Department of Physical and Analytical Chemistry, Chemistry Faculty, Academician Ye.A. Buketov Karaganda University, Karaganda 100024, Kazakhstankatsostud@mail.ru (A.K.S.); 5 Laboratory of Engineering Profile NMR, Sh. Ualikhanov Kokshetau University, Kokshetau 020000, Kazakhstan; 6 Department of Technology and Standardization, Kazakh University of Technology and Business, Astana 010000, Kazakhstan; 7 Department of Biomedicine, Karaganda Medical University, Karaganda 100012, Kazakhstan

**Keywords:** cholecalciferol, vitamin D_3_, β-cyclodextrin, nanocomposition, silver nanoparticles, encapsulation, inclusion complex, antibacterial activity, thermogravimetry

## Abstract

An important aspect of food technology is that vitamin compounds can be used for a variety of purposes, such as developing methods to enhance the nutritional value of foods. This paper discusses the synthesis and properties of β-cyclodextrin (β-CD)-functionalized silver nanoparticles, and the use of the resulting β-CD-AgNP inclusion complex when loading vitamin D_3_ (cholecalciferol, VD_3_) molecules. β-Cyclodextrin was used as a reducing agent and a stabilizer in the production of silver nanoparticles. The preparation of VD_3_-β-CD-AgNP nanocompositions was confirmed by UV spectroscopy, transmission electron microscopy, and X-ray diffraction spectroscopy. Scanning electron microscopy (SEM) and transmission electron microscopy (TEM) revealed that the resulting β-CD-VD_3_-AgNP nanocomposite was well dispersed with particle sizes ranging from 6 to 15 nm. ^1^H-, ^13^C-NMR and FTIR spectroscopy showed the reduction of silver ions and the formation of β-CD-encapsulated AgNPs. The kinetic parameters of the thermal decomposition reaction of the VD_3_-β-CD-AgNP nanocomposition have been determined under nonisothermal conditions that ensure the preservation of the kinetic triplet and a more accurate description of the process. The nanocomposition of VD_3_ with silver nanoparticles demonstrated antibacterial activity against the used bacteria.

## 1. Introduction

Today, according to the latest available medical data, the majority of the world’s population is deficient in vitamin D (VD_3_). Vitamin D deficiency is now recognized as a pandemic. Very few foods naturally contain vitamin D, and foods fortified with vitamin D are often insufficient to meet the vitamin D needs of both children and adults [1]. Modern publications indicate a number of positive effects of vitamin VD_3_ for people of all ages [1,2]. Recent studies have further clarified the role of vitamin D in the prevention of diabetes, cardiovascular diseases, cell growth, cell differentiation, embryonic development, fertility, immunological disorders, liver diseases, neurological, renal and respiratory disorders [3,4,5]. According to The World Health Organization mortality reports, vitamin D deficiency is one of the main causes of the total number of deaths (0.8 million deaths) per year [6,7,8,9]. It has been established that millions of preschool children suffer from vitamin D deficiency [10].

Achieving a normal VD_3_ supply is almost impossible without enriching the diet with vitamin D-containing products and taking cholecalciferol preparations. Vitamin D_3_, also known as cholecalciferol, includes vitamin D_2_ (ergocalciferol) and vitamin D_3_ (VD_3_). Vitamin D is responsible for improving the absorption of calcium, iron, magnesium, phosphate and zinc in the intestine, participating in metabolism [2]. However, the use of lipophilic VD_3_ as a food additive is limited by its low solubility in water and chemical instability [1,2]. In the human body, vitamin D_3_ is converted to calcifediol, and vitamin D_2_ is converted to 25-hydroxy-vitamin D_2_ [25-(OH)-D_2_]. The content of these two specific vitamin D metabolites in the blood serum determines the individual level of vitamin D. A small portion of calcifediol is converted by the kidneys into calcitriol, the biologically active form of vitamin D. Calcitriol circulates in the blood as a hormone that regulates the concentration of calcium and phosphates in the blood and promotes healthy bone growth and repair. Calcitriol also affects neuromuscular and immune functions [2,3,4]. Therefore, vitamin D_3_ has gradually become an important auxiliary substance in the food industry. The vitamin D_3_ molecule has many olefin bonds, so it is unstable in the air and easily oxidizes in a humid environment [5,6].

One of the important methods of protecting lipophilic oils is their encapsulation with starch food oligosaccharides (α-, β- and γ-cyclodextrins) (CD), which promote their uniform distribution in food raw materials [7]. Currently, β-CD is widely used in terms of increasing the solubility and chemical stability of various food additives and increasing their shelf life [7,8]. In addition, very few articles dealt with triple complexes associating β-CD, vitamin molecule, and metal ions [9]. The inclusion of VD_3_ metal complexes should allow them to be more stable in aqueous media and ensure its uniform distribution in food raw materials [9,10]. Understanding the physicochemical properties of metal nanoparticles is crucial because they influence their penetration into biological membranes and therapeutic effects. Therefore, producing silver nanoparticles (AgNPs) that are uniform in morphology and functionality is crucial for food technology applications [11]. Silver is a well-known antimicrobial agent; it is effective against bacteria, viruses, fungi, yeast, including a number of strains resistant to antibiotics [11]. The use of silver nanoparticles in the food industry is important for protecting products from microorganisms, as well as in preventing the formation of biofilms and browning of freshly cut fruits [12,13,14]. These applications are very important because it is one of the main problems in the production, transportation and storage of food [6,7,13,14,15]. In [16], a study was conducted on the inclusion of vitamin D_3_ in β-CD and triple ensembles consisting of β-CD, vitamin D_3_, and metal ions (Co(II), Cu(II), and Zn(II). In the structures of these associations, the molecular ratios of the components were 5:1:1 and 10:1:1. According to the data [2], the most optimal ratio of β-CD and vitamin D in food products is 15:1. Under these conditions, the dispersion of VD_3_ in the inclusion complex was more uniform, while the stability and absorption rate of the inclusion complex were significantly higher than those of the initial VD_3_.

In previous study, we described the preparation and some features of vitamin VD_3_ encapsulation using β-CD [6]. In the present study, we have demonstrated the preparation and thermochemical characterization of encapsulated β-CD complexes incorporating vitamin VD_3_ as an organic ligand with AgNPs. Modified vitamin VD_3_-β-CD-AgNP compositions can be considered a promising platform for improving stability and expanding their potential in food products. The inclusion of VD_3_ metal complexes should allow them to be more stable in aqueous environments. Future research should test these hypotheses.

## 2. Materials and Methods

### 2.1. Reagents and Synthetic Procedures

The following reagents were used: *β*-Cyclodextrin (99%) (Aldrich, Munich, Germany, mp 270–290 °C with decomp.); Cholecalciferol (VD_3_) (Sigma, Houston, TX, USA), “analytically grade”. Silver nitrate (AgNO_3_) and NaOH hydroxide were purchased from Sinopharm Chemical Reagent Co., Ltd. (Shanghai, China). All solutions were prepared with Elga Millipore deionised water. All glassware used in the experiments was washed with a fresh HNO_3_/HCl solution (3:1 by volume) before use, then thoroughly rinsed with bidistilled water. All reagent solutions were prepared with bidistilled water. The UV absorption spectra of β-CD-AgNP solutions were recorded using an N60 Implen UV-visible spectrophotometer (Radnor, PA, USA) (200–600 nm). ^1^H, ^13^C NMR spectra were obtained using a JNN-ECA 400 instrument Jeol (Tokyo, Japan) using D_2_O and DMSO-d_6_ as a solvent. Chemical shifts are measured relative to the signals of residual protons or carbon atoms of the deuterated solvent. The FTIR spectrum was measured using a Nicolet iS50 external FTIR spectrometer (Thermo Scientific, Waltham, MA, USA) (KBr, 99%). X-ray imaging was obtained on an XD6 X-ray diffractometer at 40 kV and 30 mA with a scan rate of 5 °/min and a scan range of 20–90° using Cu Ka radiation (l = 0.1546 nm). Samples were analyzed using a JEM-1400 transmission electron microscope (Tokyo, Japan) at an accelerating voltage of 80 kV. Samples for TEM were prepared by applying a few drops of freshly prepared sol to the surface of a copper grid coated with a carbon support film and drying at room temperature. The particle size distribution was obtained using a TEM image using ImageJ software (1.50d, Hitachi 7600 TEM, Hitachi, Tokyo, Japan). The surface morphology of the nanoparticles was determined using a Tescan Mira 3LMN scanning electron microscope (Prague, Czech Republic). The samples were attached to a conductive adhesive surface and observed at an accelerating voltage of 15 kV. The shape, size, and distribution of AgNPs were examined using a transmission electron microscope (TEM). Nanocomposite samples were prepared by applying a few drops of the prepared sol to the surface of a copper grid coated with a carbon carrier film and drying at room temperature. TEM was performed on a JEM-1400 transmission electron microscope at an accelerating voltage of 80 kV. The nanoparticle size distribution was obtained using ImageJ software.

### 2.2. Preparation of the VD_3_–β-CD–AgNP Inclusion Complexes

VD_3_–β-CD–AgNPs was synthesized by in situ reduction according to the described methods [16,17,18,19,20] with minor modifications. The method described here involved in the first step the reduction of the [Ag(NH_3_)_2_]^+^ complex to metallic Ag^0^ with an aqueous solution of β-CD. 5 mL of β-CD solution (0.1 M) was added to 30.0 mL of water, to which NH_4_OH (10%) was gradually added with stirring until pH reached 9.

**Figure 1:**
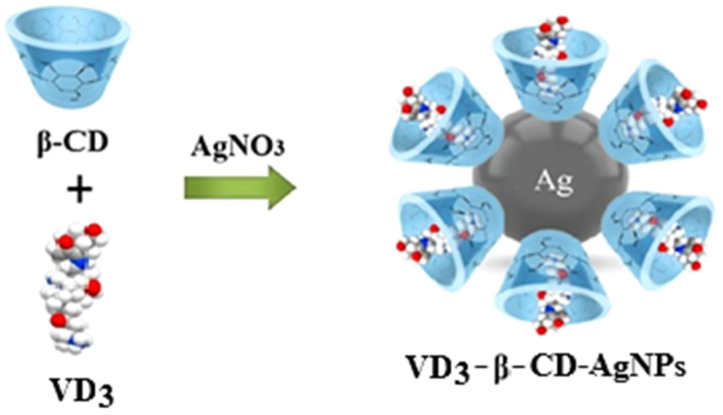
Schematic representation for the synthesis of VD_3_–β-CD–AgNPs.

**Figure 2:**
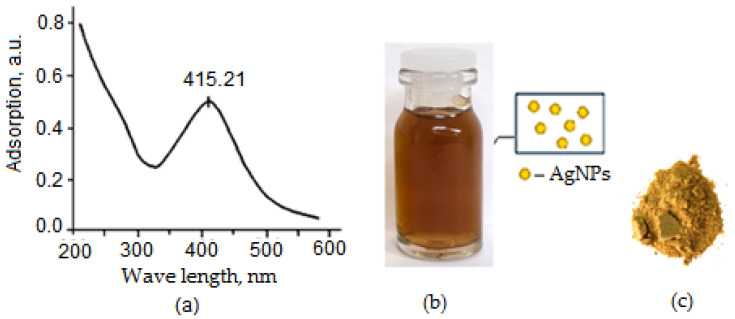
UV-visible spectrum of VD_3_–β-CD–AgNPs (**a**), color of VD_3_–β-CD–AgNP solution under optimal conditions (**b**) and dry powder VD_3_–β-CD–AgNPs (**c**).

5 mL of AgNO_3_ solution (0.001 M) was added dropwise to the resulting solution and the reaction was carried out for 2 h at 70 °C until a yellow-brown solution of β-CD–AgNPs was obtained. As the reaction progressed, the color of the solution changed to an intense yellow-brown, indicating the formation of AgNP nanoparticles (Figure 1 and Figure 2a–c). This may be associated with the excitation of surface plasmon oscillation of AgNPs, which is consistent with literature data [21,22].

This may be associated with the excitation of surface plasmon oscillations of AgNPs, which is consistent with literature data [21,22]. Increasing the reaction time and pH of the solution resulted in the formation of a dark brown and then gray solution with precipitation of metallic silver. These observations show that with the increase in reaction time, the particle size and aggregation of silver nanocrystals gradually increase. All measurements were carried out at room temperature (20 ± 0.05 °C). The resulting yellow-brown β-CD–AgNP solution was used to encapsulate VD_3_ molecules then. The VD_3_–β-CD–AgNPs (15:1:1) complex was prepared by slowly adding 5 mL of an acetone solution of VD_3_ (0.001 M) to an aqueous solution of β-CD–AgNPs. Each experiment was repeated three times. The synthesis scheme of VD_3_–β-CD–AgNPs is shown in Figure 1.

The VD_3_ encapsulation process was carried out with constant stirring of the solution for 24 h at room temperature for slow and complete evaporation of acetone. Each experiment was repeated three times. Supramolecular assemblies were dried in a rotary evaporator under vacuum until the solids were completely dry (Figure 2a–c). Finally, they were stored in small sealed glasses in a desiccator. The absorption spectrum of β-CD–VD_3_–AgNPs in the UV range at 415.21 nm indicates that the obtained nanoparticles have an absorption peak characteristic of spherical nanoparticles (Figure 2a) [19,20,21,22].

Thermal decomposition of the β-CD-Ag (15:1) and VD_3_–β-CD-Ag (15:1:1) inclusion complexes was performed on a Labsys Evo TG-DTA/DSC differential thermal analyzer (SETARAM, Caluire-et-Cuire, France) in corundum crucibles in the temperature range from 30 up to 1000 °C in an inert gas. The consumption of protective and purge gas was 20 and 50 mL/min, respectively. The heating rate of the samples was 10.0 °C/min. The mass of each sample used for the thermal decomposition study was 10.0 ± 0.5 mg. Each measurement was performed three times to ensure reproducibility of the results and subsequent statistical averaging. Experimental data were processed and plotted using OriginPro 9.0 and Anaconda (Python 3.10) with NumPy, SciPy, and Matplotlib 3.10.1 libraries.

### 2.3. Bacteria and Cultivation Conditions

The work used strains of *E. coli* K-12 (laboratory strain), *S. aureus* MDC5233 and *E. hirae* ATCC9790. The bacteria were grown under anaerobic conditions on a peptone medium (2% peptone, 0.5% NaCI, 0.2% K_2_HPO_4_ and 0.2% glucose) at 37 °C and pH 7.5 [17]. To maintain anaerobic conditions and maintain O_2_ levels, the vessels were kept hermetically sealed. The bacterium was cultured in 250 mL vessels with tightly closed airtight stoppers. For sowing, 1.5% of a pre-grown liquid bacterial culture was used. Bacterial growth was determined by the method [17,18] by measuring the optical density of suspensions on a UV-1280 spectrophotometer (Shimadzu, Japan) in a cuvette with an optical path length of 10 mm at a wavelength of 600 nm.

The growth rate (*v*) was determined by the formula

$$v\;=\;\frac{\mathrm{In}D_{t}\;-\;\mathrm{In}D_{0}}{t},$$

*v* = where D_0_ is the initial value of the optical density (D_600_), and D_t_ is D_600_ after time t.

This value was expressed in hours to the power of minus one. Viable bacteria were counted by inoculating a correspondingly diluted bacterial culture onto a solid nutrient medium in Petri dishes and then quantifying the resulting colonies.

### 2.4. Statistical Analysis

All values in this study were presented as mean ± SE of at least 3 independent experiments. Two-way ANOVA for significance testing was used for multiple group analysis and Student’s *t*-tests were used for two-group comparison, *p*-value < 0.05 was considered as significant.

## 3. Results and Discussion

### 3.1. Characteristics of the Structure of VD_3_-β-CD-AgNP Nanocomposites

The dispersion of the resulting β-CD-AgNP nanocomposites with encapsulated vitamin VD_3_ was studied using transmission electron microscopy (TEM). Analysis of the morphology and dispersion of the obtained data revealed the various sizes and spherical nature of the resulting nanoparticles. The histogram revealed a narrow particle size distribution ranging from 6 to 20 nm, with an average size of 9.25 ± 1.17 nm.

**Figure 3:**
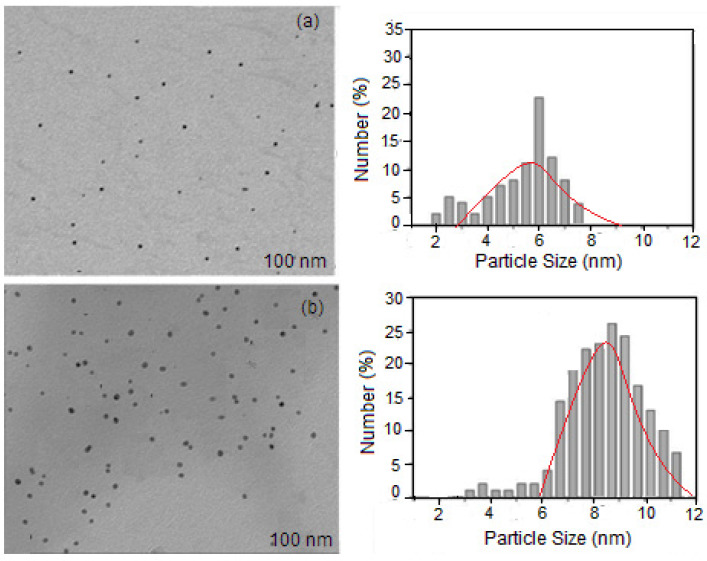
TEM images and the time evolution of the size distribution of the AgNP nanoparticles. The average particle sizes are (**a**) 5.6 nm, 40 min, pH = 9.13; (**b**) 6.70 nm, 60 min, pH = 9.65.

**Figure 4:**
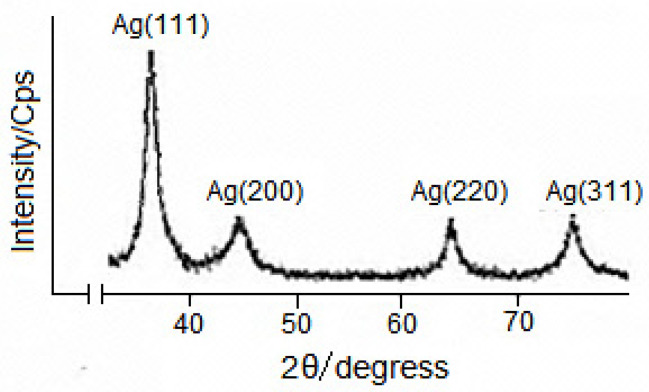
X-ray images of AgNPs, the resulting nanocomposite with VD_3_–β-CD–AgNPs.

The histograms clearly show an increase in particle size with increasing time. The observed pattern indicates a slow growth of AgNP nanoparticles in the β-CD matrix. Analysis of the temporal evolution of the particle size distribution also suggests a clear size selectivity of the reaction, and the preferred particle sizes appear to be about 7–11 nm (Figure 3). In nanoparticle production studies, controlling the size, shape, and morphology of AgNPs is crucial. X-ray diffraction is a key tool for studying nanoscale particles [22,23]. Figure 4 shows X-ray images of the AgNPs in the obtained VD_3_-β-CD-AgNP nanocomposite (Figure 4). Analysis of the obtained results indicates the formation of a crystalline structure of silver nanoparticles. The XRD peaks in a wide range of 2° angles (30° < 2° < 80°) showed that the peaks at 38.02°, 44.07°, 64.35° and 77.21° could be assigned to 111, 200, 220 and 311 crystal structures of face-centered cubic (FCC) silver nanocrystal, respectively (Ag XRD Ref. No. 00-004-0783) [20,21,22,23,24]. X-ray diffraction a modern technique used mainly to determine the state of a substance (crystalline or amorphous) under different irradiation angles. No other peaks present as impurities were found on the X-ray images. Thus, these results provide clear evidence of the presence of AgNPs in the VD_3_–β-CD–AgNP composition.

**Figure 5:**
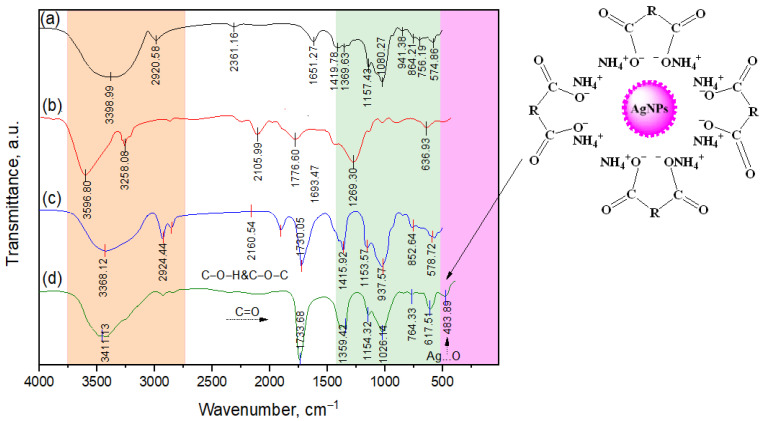
Fourier transform IR spectra of β-CD (**a**), VD_3_ (**b**), β-CD–AgNPs (**c**) and VD_3_–β-CD-AgNPs (**d**).

The formation of VD_3_–β-CD–AgNPs was also confirmed by FT-IR spectroscopy (Figure 5). In the spectra of β-CD (a), β-CD–AgNPs (c), and VD_3_–β-CD-AgNPs (d), the stretching vibrations of the OH bond appear as a broad band with a maximum at 3411, 3398, and 3368 cm^−1^ in all systems. There are also absorption bands at 2924 and 2920 cm^−1^, characteristic of the stretching vibrations of C–H bonds in the CH and CH_2_ groups [20,22].

The absorption bands at 1651–1730 cm^−1^ are characteristic of the stretching vibrations of the C=O bond, and the absorption bands at 1415 and 1359 cm^−1^ are due to the deformation vibrations of the C–H bonds in the CH_2_OH and CHOH groups. The absorption bands of C=C bonds and other cholecalciferol groups are not visible in the IR spectra of the beta-CD-VD_3_ complex (Figure 5) [23,24]. This may mean that these groups are masked by the very broad and intense absorption bands of β-CD in the same wavelength range. The formation of the β-CD–AgNPs, VD_3_–β-CD and VD_3_–β-CD–AgNP nanocomposition was also studied using ^1^H, ^13^C NMR and COSY, HMQC, HMBC spectroscopy (Figures S1–S7). The ^1^H and ^13^C NMR chemical shift values of β-CD in a free and complexing state are shown in Table 1. All six β-CD protons show a pronounced chemical shift towards a strong field. The largest difference in the chemical shift values Δδ in the β-CD–VD_3_^1^H NMR spectrum is characteristic for the H-3 (0.115 м.д.) and H-5 (0.117 м.д.) intraspheric protons [25,26]. It should be noted that the nature of the manifestation of absorption bands OH and C=O groups in the spectrum of the VD_3_–β-CD–AgNP complex is influenced by the screening effect of the interaction of AgNPs with β-CD.

### 3.2. Thermogravimetric Analysis of β-CD–AgNP, VD_3_–β-CD and VD_3_–β-CD–AgNP

**Figure 6:**
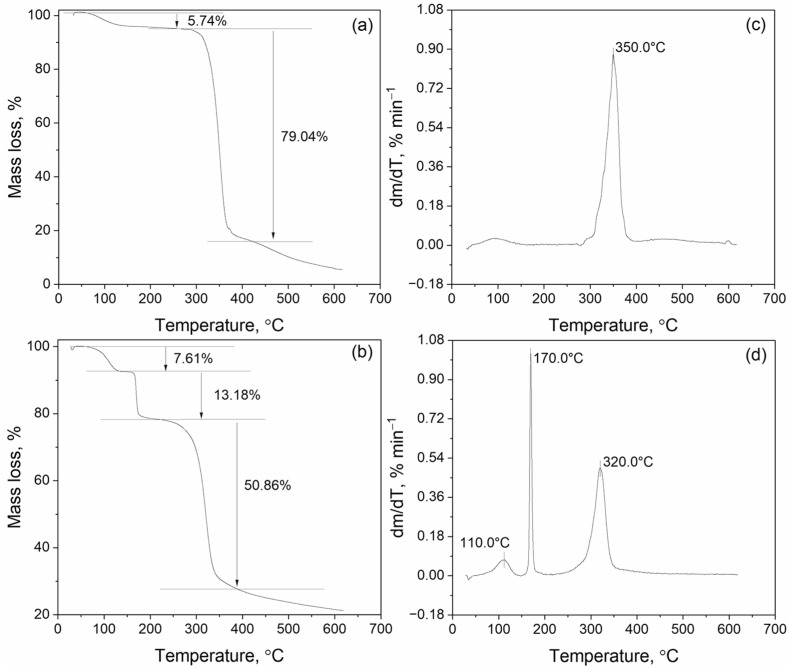
Thermogravimetric (TG) and differential thermogravimetric (DTG) curves of thermal decomposition of inclusion complexes β-CD–Ag (15:1) (**a**,**c**) and VD_3_–β-CD–Ag (15:1:1) (**b**,**d**) inclusion complexes.

Thermogravimetric (TG) and differential thermogravimetric (DTG) analyses were performed to study the thermal decomposition of β-CD-AgNPs (15:1) and β-CD–VD_3_–AgNP (15:1:1) complexes. TG curves (Figure 6a,b) demonstrate a stepwise decrease in the mass of samples with increasing temperature, which reflects the multi-stage process. The corresponding DTG curves (Figure 6c,d), which are derived from TG, display the decomposition rate as a function of temperature (Figures S8 and S9). For the β-CD–Ag complex (Figure 6a,c), one pronounced DTG peak is observed, indicating a predominantly one-step mechanism. The first stage (up to ~120 °C) is accompanied by a mass loss of 5.74% by removing adsorbed and crystallized water. The most intense decomposition of the complexes occurs in the range of ~250–400 °C with a mass loss of up to 79.04%, which is associated with the destruction of the outer shell of the β-CD structure. The VD_3_–β-CD–Ag complex (Figure 6b,d) shows a more complex decomposition pattern, including three stages: stage I (up to ~130 °C)—mass loss of 7.61% associated with the removal of moisture from the β-CD matrix; stage II (~130–240 °C)—mass loss of 13.18%, due to the initial destruction of β-CD and partial decomposition of VD_3_.

At this stage, the decomposition rate of the composition is not yet at its maximum. At stage III (~240–420 °C), the main mass loss of the sample is observed with a maximum on the DTG curve, corresponding to deep degradation of β-CD and coordination products. A comparison of thermograms shows that the addition of VD_3_ reduces the temperature at the beginning of decomposition and increases the multi-stage process. This indicates a modification of the degradation mechanism and the involvement of VD_3_ in the formation of a stable complex.

**Figure 7:**
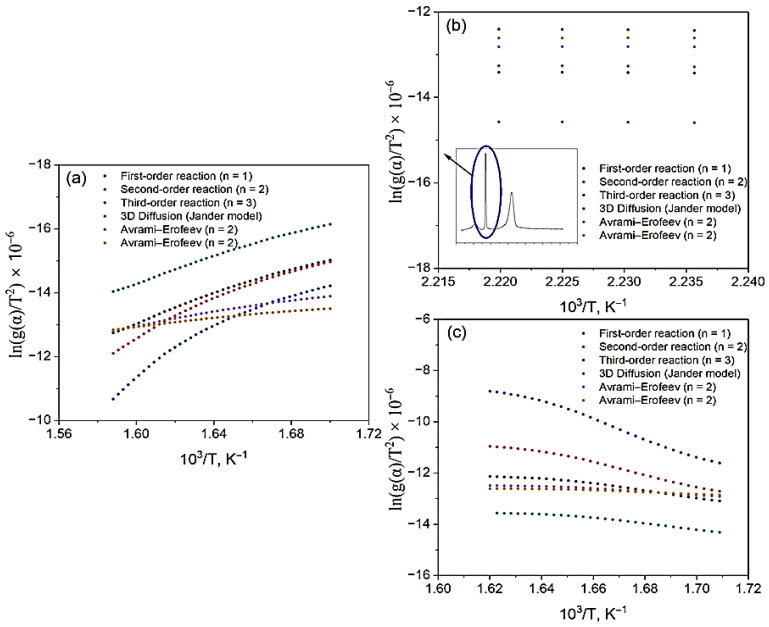
Graphical representation of the dependence of ln[g(α)/T^2^] on 1/T, calculated using the Coates–Redfern method, for the complex (**a**) β-CD–AgNPs (10:1) and (**b**) VD_3_–β-CD–AgNPs (10:1:1) using various kinetic models. (**c**) VD3–β-CD–AgNPs in the area corresponding to the second peak of the DTG curve.

The kinetic parameters were calculated using the Coates–Redfern method [27] using various models: first-, second-, and third-order reactions, the three-dimensional diffusion model (the Yander model), and the Avrami–Yerofeyev models [28] with *n* = 2 and *n* = 3 growth rates for the nucleation of a new phase (or new structure), during the decomposition of the sample. The vertical segments on the graphs reflect the standard deviations obtained as a result of a linear approximation of the dependence ln[g(α)/T^2^] on 1/T, plotted for each value of the degree of transformation α (Figure 7a).

**Figure 8:**
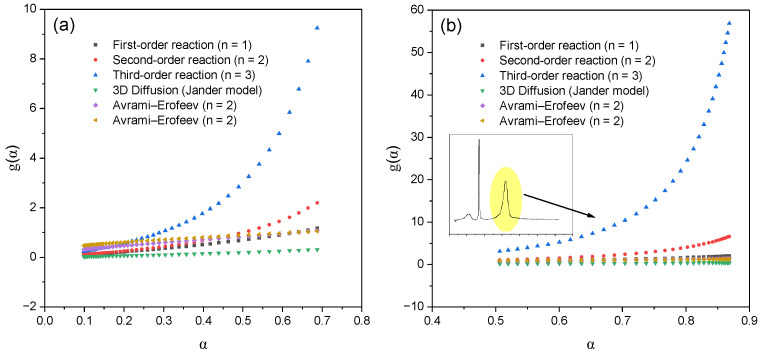
Dependences of the integral transformation functions g(α) on the degree of transformation α for various kinetic models applied to the thermal decomposition of (**a**) β-CD–AgNP (15:1) and (**b**) VD_3_–β-CD–AgNP (15:1:1) complexes.

In Figure 7b, the temperature and α-range corresponding to the first peak of the DTG curve are highlighted, in which an approximation was performed to calculate the kinetic parameters. This range is characterized by a high degree of linearity of dependencies for most models, which confirms the reliability of the obtained values. The exception is the first-order model, for which deviations from linearity are observed, indicating its limited applicability to describe kinetics in this temperature range. Figure 7c shows the region corresponding to the second peak of the DTG curve, within which ln[g(α)/T^2^] from 1/T was also linearly approximated. This area reflects the main stage of thermal decomposition, characterized by the maximum rate of destruction. The calculations performed made it possible to obtain representative and reliable values of the activation energy and the pre-exponential multiplier, specific for this stage of decomposition. A comparison between the complexes showed that for VD_3_–β-CD–AgNPs, the activation energy values are lower in all models compared with β-CD–AgNPs, which may be due to a change in the mechanism of thermal destruction and partial stabilization of the complex structure due to the inclusion of vitamin D_3_. Figure 8 shows the dependences of the integral transformation functions g(α) on the degree of transformation α for various kinetic models applied to the thermal decomposition of (a) β-CD–AgNP (15:1) and (b) VD_3_–β-CD–AgNP (15:1:1) complexes.

On these dependences, the degree of trnsformation α (in the range from 0 to 1) is plotted along the abscissa axis, and the values of the corresponding integral functions g(α), characteristic of six classical kinetic models: reactions of the first, second, and third orders, as well as the Yander and Avrami–Yerofeyev models [28] of three-dimensional diffusion, are plotted along the ordinate axis, *n* = 2 and at *n* = 3.

**Figure 9:**
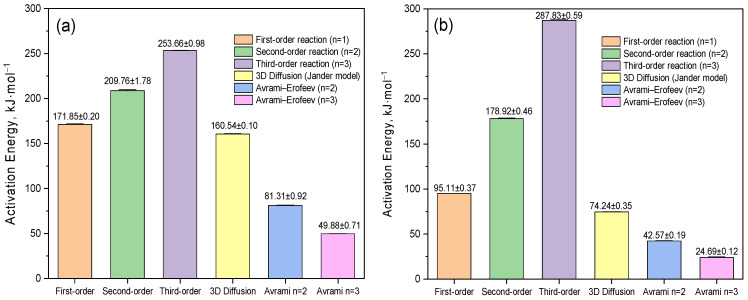
(**a**) Comparative analysis of activation energies and (**b**) corresponding errors for various kinetic mechanisms applied to the thermal decomposition of β-CD–Ag (15:1) and VD_3_–β-CD–Ag (15:1:1) complexes.

Each curve reflects the behavior of the function g(α) used in the traditional Coates–Redfern method [27] for calculating kinetic parameters. The graphs allow you to visually compare how different the integral functions are between the models for different values of α. Figure 8b shows that the characteristic range of the degree of transformation corresponding to the second peak on the differential thermogram (da/dT) is highlighted in yellow. The parameters calculated in this range relate to the second stage of thermal decomposition, which differs from the first in kinetic characteristics. This indicates a multi-stage decomposition mechanism, each stage of which requires an individual approach to interpretation and model selection. At low values of α (0.1–0.3), most models show similar values of g(α), which indicates a low sensitivity of the model selection at the initial stage of decomposition. On the contrary, at high degrees of transformation (α ≈ 0.6–0.9), the behavior of the functions diverges significantly, and the correct choice of the kinetic model becomes critically important for the accurate calculation of the activation energy and the pre-exponential multiplier. It is worth noting separately that the Avrami–Yerofeyev models at *n* = 2 and *n* = 3 demonstrate a smooth, nonlinear increase in g(α), typical for autocatalytic and embryo growth processes [28,29,30,31]. At the same time, the second- and third-order models are characterized by a sharp increase at α→1, which corresponds to mechanisms with an accelerating reaction course. Thus, this graph allows us to determine the ranges α in which any model can be applied without significant error, as well as critical areas in which the choice of model has a significant impact on the results. This is especially relevant for step-by-step kinetic analysis, as in the case of decomposition of the VD_3_-β-CD-AgNP complex. The activation energy values shown in Figure 9 were determined using the Coates–Redfern method [27] to analyze various kinetic models, including first-, second-, and third-order reactions, the Yander three-dimensional diffusion model, and the Avrami–Yerofeyev model (*n* = 2.3) [28,29,30,31].

The left panel (a) illustrates the energy characteristics of the β-CD–AgNP complex (15:1), while the right panel (b) reflects the corresponding values for the VD_3_–β-CD–Ag complex (15:1:1). Each column in Figure 9 is the average value of the activation energy with an indication of the standard deviation calculated based on a linear approximation of the dependence ln[g(α)/T^2^] on 1/T corresponding to a specific kinetic decomposition model. The mentioned dependence, as well as the features of its application, were discussed in detail above and visualized in Figure 8, which shows the behavior of integral functions g(α) used to determine kinetic parameters by the Coats-Redfern method [27]. The highest activation energy values are observed when using third- and second-order models, which suggests the accelerated nature of the process in the late stages of decomposition. At the same time, significantly lower values obtained using the Avrami–Yerofeyev models [28,29,30,31] may indicate control of the process by autocatalytic mechanisms or the formation and growth of embryos.

### 3.3. Antibacterial Activity

**Figure 10:**
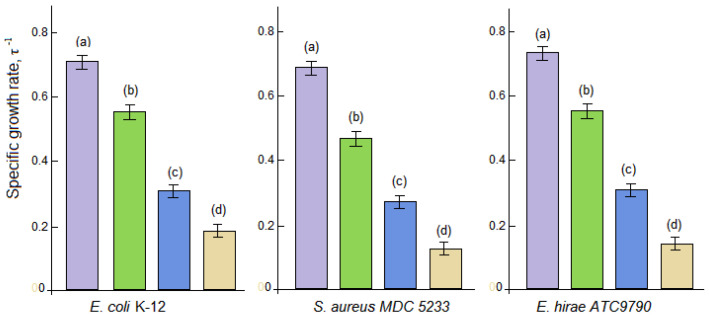
The dependence of the specific growth rate of Gram-negative and Gram-positive bacteria on the concentration of nucleic acids. (**a**)—control without NC; (**b**)—bacterium + NC (30 μg/mL); (**c**)—bacterium + NC (40 μg/mL); (**d**)—bacterium + NC (50 μg/mL).

The effect of the concentration of the nanocomposite (NC) VD_3_–β-CD–AgNPs (15:1:1) the specific growth rate of *Gram*-negative (*Escherichia coli* K-12) and *Gram*-positive bacteria (*Enterococcus hirae*, *Staphylococcus aureus* MDC5233), which are widely used in microbiological research as model microorganisms was studied [17,18]. The VD_3_–β-CD–AgNP nanocomposite exhibited antibacterial activity against the bacteria used, suppressing their specific growth rate (Figure 10). The maximum inhibitory effect of VD_3_–β-CD–AgNPs was observed at a concentration of 50 μg/mL. At the same time, the activity of VD_3_–β-CD-AgNP nanocompositions on *S. aureus* and *E. hirae* was more pronounced than on *E. coli*. Under the conditions studied, the specific growth rate of *E. coli* slowed down by about 4.0 ± 0.3 times, while the growth of *S. aureus* and *E. hirae* was suppressed by 6.0 ± 0.5 and 6.5 ± 0.2 times, respectively. From the analysis of the data obtained, it follows that Gram-negative bacteria turned out to be more resistant to the action of VD_3_–β-CD–AgNP nanoparticles than Gram-positive ones. This result may be related to the structure of the bacterial cell wall and the presence of an outer membrane that serves as a barrier for nanocompositions. According to literature data [32,33,34,35], the antibacterial activity of Ag particles may be the result of the appearance of free positively charged Ag^+^ ions, their adsorption on the negatively charged surface of the bacterial membrane, as well as their penetration into the cell. In addition, free Ag^+^ ions can contribute to the formation of reactive oxygen species in the physiological environment, which can lead to oxidative stress, damage to protein structures and cell membranes, and bacterial death. The effect of VD_3_–β-CD–AgNPs on the colony-forming units (CFU) of bacterial samples has been studied. When adding 50 micrograms/mL of nanocomposition, the number of viable colonies of *E. coli*, as well as *S. aureus* and *E. hirae*, grown on a dense nutrient medium decreased by 48 ± 0.3, 60 ± 0.2 and 67 ± 0.2%, respectively, compared with the control (without nanoparticles), which indicated the bactericidal effect of the nanocompositions. The data were calculated based on three parallel experiments. Colony Forming Unit (CFU) is a microbiological term used to estimate the number of viable bacteria or fungal cells in a given sample.

## 4. Conclusions

The results of this study demonstrate the production of a water-soluble complex of vitamin D_3_ molecules with functionalized β-cyclodextrin by silver nanoparticles to form a VD_3_–β-CD–AgNP inclusion complex in a molar ratio of 15:1:1. FT-IR, DSC, TGA and X-ray diffraction methods have shown that the VD_3_–β-CD–AgNP nanocomposition has physico-chemical characteristics other than nonencapsulated VD_3_ and β-CD–AgNPs. The differences obtained emphasize the effect of the inclusion of AgNPs on the thermal stability of the supramolecular system. The bactericidal effect of the VD_3_–β-CD–AgNP inclusion complex on Gram-negative *E. coli* K-12, Gram-positive *S. aureus* MDC5233 and *E. hirae* ATCC9790 was revealed. Under the conditions studied, Gram-negative bacteria turned out to be more resistant to the effects of nanocomposition, which may be due to the presence of their outer membrane, which serves as a kind of barrier for silver nanoparticles.

## Figures and Tables

**Table 1:** ^1^H and ^13^C NMR chemical shifts (Δδ) of β-CD in a free state and as a part of the β-CD-VD_3_ inclusion complex.

Atom Number	β-CD (δ_0_) (ppm)		β-CD-VD_3_ (δ) (ppm)		Δδ (δ-δ_0_) (ppm)	
	δ (^1^H)	Δ (^13^C)	δ (^1^H)	Δ (^13^C)	δ (^1^H)	Δ (^13^C)
C1	4.823	102.43	4.736	102.23	−0.087	−0.18
C2	3.543	72.85	3.455	72.81	−0.078	−0.04
C3	3.510	73.55	3.395	73.43	−0.115	−0.12
C4	3.477	82.15	3.439	82.08	−0.038	−0.07
C5	3.352	72.51	3.235	72.39	−0.117	−0.12
C6	3.630	60.37	3.547	60.24	−0.083	−0.13

## Data Availability

The original contributions presented in the study are included in the article and Supplementary Material, further inquiries can be directed to the corresponding authors.

## Supplementary Materials

The following supporting information can be downloaded at https://www.mdpi.com/article/10.3390/molecules30244823/s1, Figure S1. ^1^H NMR spectrum of β-CD (DMSO); Figure S2. COSY (^1^H-^1^H) NMR spectrum of β-CD (DMSO); Figure S3. HSQC (^1^H-^13^C) NMR spectrum of β-CD (DMSO); Figure S4. HMBC NMR spectrum (^1^H-^13^C) of β-CD (DMSO); Figure S5. ^1^H (a), ^13^C (b) NMR spectra of β-CD-AgNPs (D_2_O); Figure S6. (a–c) COSY (a), HMQC (b), HMBC (c) spectra of β-CD-AgNPs, (D2O); Figure S7. (a,b) ^1^H (a) and ^13^C(b) NMR spectra of VD_3_ (DMSO); Figure S8. (a,b) Graphical dependences of mass change on temperature: (a) β-CD–Ag (15:1) and (b) VD_3_–β-CD–Ag (15:1:1); Figure S9. Kinetic curves of the dependence of the degree of transformation on time during thermal decomposition of β-CD–Ag (15:1) (a) and VD_3_–β-CD–Ag (15:1:1) (b).

## References

[1] Holick M.F., Chen T.C. (2008). Vitamin D deficiency: A worldwide problem with health consequence. Am. J. Clin. Nutr..

[2] Liu Y., Zhang H. (2016). Study of VD_3_-β-Cyclodextrin Inclusion Complex. J. Geosci. Environ. Prot..

[3] Mauryaa V.K., Bashirb K., Aggarwala M. (2020). Vitamin D microencapsulation and fortification: Trends and technologies. J. Steroid Biochem. Mol. Biol..

[4] Harmon Q.E., Umbach D.M., Baird D.D. (2016). Use of Estrogen-Containing Contraception Is Associated With Increased Concentrations of 25-Hydroxy Vitamin. J. Clin. Endocrinol. Metab..

[5] Bischoff-Ferrari Heike A., Willet Walter C., Wong John B., Giovannucci E., Dietrich T., Dawson-Hughes B. (2005). Fracture Prevention with Vitamin D Supplementation. Am. Medic. Assoc..

[6] Bakirova R., Nukhuly A., Iskineyeva A., Fazylov S., Burkeyev M., Mustafayeva A., Minayeva Y., Sarsenbekova A. (2020). Obtaining and Investigation of the β-Cyclodextrin Inclusion Complex with Vitamin D_3_ Oil Solution. Scientifica.

[7] Legarth C., Grimm D., Wehland M., Bauer J., Kruger M. (2018). The impact of vitamin D in the treatment of essential hypertension. Int. J. Mol. Sci..

[8] Astray G., Gonzalez-Barreiro C., Mejuto J.C., Rial-Otero R., Simal-Gándara J. (2009). A Review on the Use of Cyclodextrins in Foods. Food Hydrocoll..

[9] Szejtli J., Bolla-Pusztai E., Szabo P., Ferenczy T. (1980). Enhancement of Stability and Biological Effect of Cholecalciferol by β-Cyclodextrin Complexation. Pharmazie.

[10] Merce A.L.R., Yano L.S., Khan M.A., Thanh X.D., Bouet G. (2003). Complexing Power of Vitamin D3 Toward Various Metals. Potentiometric Studies of Vitamin D3 Complexes with AI^3+^, Cd^2+^, Gd^3+^, and Pb^2+^ Ions in Water-Ethanol Solution. J. Solut. Chem..

[11] Biswas P.K., Dey S. (2015). Effects and applications of silver nanoparticles in different fields. Int. J. Recent Sci. Res..

[12] Bajpai V.K., Kamle M., Shukla S., Mahato D.K., Chandra P. (2018). Prospects of using nanotechnology for food preservation, safety, and security. J. Food Drug Anal..

[13] He X., Hwang H.M. (2016). Nanotechnology in food science: Functionality, applicability, and safety assessment. J. Food Drug Anal..

[14] Carbone M., Domencia D., Sabbatella G., Antiochia R. (2016). Silver nanoparticles in polymeric matrices for fresh food packaging. J. King Saud Univ. Sci..

[15] Camberos E.P., Hernandez I.M.S., Aguirre S.I.J., Garcia J.J.O., Cervantes C.M. (2019). Food Industry Applications of Phyto-Synthesized Silver Nanoparticles. Glob. J. Nutri. Food Sci..

[16] Merce R.A.L., Nicolini J., Khan M.A., Bouet G. (2009). Qualitativ study of supramolecular assamblies of beta-cyclodextrin and cholecalciferl and the cobalt (II), copper (II) and zinc (II) ions. Carbohydr. Polym..

[17] Gabrielyan L., Hovhannisyan A., Gevorgyan V., Ananyan M., Trehounian A. (2019). Effect of iron oxide (Fe_3_O_4_) nanoparticles: Distinguishing concentration-dependent effects with different bacterial cells grouth and membrane-associated mechanisms. Appl. Microbiol. Biothechnol..

[18] Gabrielyan L., Nakbyan L., Hovhannisyan A., Trchounian A. (2019). Effects of iron oxide (Fe_3_O_4_) nanoparticles on *Escherichia coli* antibiotic-resistant strains. J. Appl. Microbiol..

[19] Yang X.X., Li C.M., Huang C.Z. (2016). Curcumin modified silver nanoparticles for highly efficient inhibition of respiratory syncytial virus infection. Nanoscale.

[20] Soares D.F., Noseda M.D., Felcman J., Khan M.A., Bouet G., Mercê A.L.R. (2013). Supramolecular assemblies of Al^3+^ complexes with vitamin D_3_ (cholecalciferol) and phenothiazine. Encapsulation and complexation studies in β-cyclodextrin. J. Incl. Phenom. Macrocycl. Chem..

[21] Yang K., Liu J., Luo L., Li M., Xu T., Zan J. (2023). Synthesis of cationic b-cyclodextrin functionalized silver nanoparticles and their drug-loading applications. RSC Adv..

[22] Ze H., Yan Sun X., Jing Y., Zhang J., Li X., Zhang H., Shakoor A., Guo J. (2022). UV-irradiation synthesis of cyclodextrin-silver nanocluster decorated TiO_2_-nanoparticles for photocatalytic enchanced anticancer effect on Hela cancer cells. Front. Chem..

[23] Zargar M., Hamid A., Bakar F.A., Shamsudin M.N., Shameli K., Jahanshiri F., Farahani F. (2011). Green Synthesis and Antibacterial Effect of Silver Nanoparticles Using *Vitex negundo* L.. Molecules.

[24] Shameli K., Ahmad M.B., Jazayeri S.D. (2012). Investigation of antibacterial properties silver nanoparticles prepared via green method. Chem. Cent. J..

[25] Novakowski M., Ejchart A. (2014). Complex formation of fenchone with α-cyclodextren: NMR titrations. J. Incl. Phenom. Macrocycl. Chem..

[26] Maheshwari A., Sharma M., Sharma D. (2013). Complexation of sodium picosulphate with beta cyclodextri: NMR spectroscopic study in solution. J. Incl. Phenom. Macrocycl. Chem..

[27] Coats A.W., Redfern J.P. (1964). Kinetic parameters from thermo-gravimetric data. Nature.

[28] Avrami M. (1941). Granulation, Phase Change, and Microstructure Kinetics of Phase Change II. J. Chem. Phys..

[29] Vardanyan Z., Gevorkyan V., Ananyan M., Vardapetyan H., Trchounian A. (2015). Effect of various heavy metal nanoparticles on *Enterococcus hirae* and *Escherichia coli* growth and proton-coupled membrane transport. J. Microbiol..

[30] Wang G., Zhang J., Shao J., Liu Z., Zhang G., Xu T., Guo J., Wang H., Xu R., Lin H. (2016). Thermal behavior and kinetic analysis of co-combustion of waste biomass/low rank coal blends. Energy Convers. Manag..

[31] Li G.S., Yong G.P., Yan X.Y., Hu Y. (2003). Comparison to the thermal decomposition kinetics of several inclusion complex of b-cyclodextrin. Chem. Res. Appl..

[32] Wang L., Hu C., Shao L. (2017). The antimicrobial activity of nanoparticles: Present situation and prospects for the future. Int. J. Nanomed..

[33] Burdusel A.C., Gherasim O., Grmezescu A.M., Mogoanta L., Ficai A., Andronescu E. (2018). Biomedical applications of silver nanoparticles: An up-to-date overview. Nanomaterials.

[34] Lee S.H., Jun B.H. (2019). Silver nanoparticles: Synthesis and application for nanomedicine. Int. J. Mol. Sci..

[35] MsShan D., Ray P.S., Yu H. (2014). Molecular toxicity mechanism of nanosilver. J. Food Drug Anal..

