# Supplementary material for: Preparation of a Supramolecular Assembly of Vitamin D in a β-Cyclodextrin Shell with Silver Nanoparticles
Source: Molecules. 2025 Dec 18;30(24):4823. doi: 10.3390/molecules30244823 (PMC12735546; doi:10.3390/molecules30244823)
Supplement: Supplementary file 1 [file molecules-30-04823-s001.zip › molecules-3989247-supplementary.pdf]

Preparation of silver nanoparticles functionalized with β-cyclodextrin and their use for loading vitamin D molecules

Ryszhan Ye. Bakirova[1], Serik D. Fazylov[*2], Ainara S. Iskineyeva[3], Akmaral Zh. Sarsenbekova[4], Aleksandr K. Sviderskiy[4], Olzhas T. Seilkhanov[5], Ayaulym K. Mustafayeva[*3], Anel Zh. Mendibayeva[2], Bolatkul Dh. Ashirbekova[1], Mereke T. Agedilova[6], Gaukhar Khabdolla[7]

[1] Department of Internal Diseases, Karaganda Medical University, 100012, Karaganda, Kazakhstan
[2] Institute of Organic Synthesis and Coal Chemistry, 100008, Karaganda, Kazakhstan
[3] Department of Technology of Food and Processing Industries, Saken Seifullin Kazakh Agrotechnical University, 010000, Astana, Kazakhstan
[4] Department of Physical and Analytical Chemistry, Chemistry Faculty, Academician Ye.A. Buketov Karaganda University, 100024, Karaganda, Kazakhstan
[5] Laboratory of Engineering Profile NMR, Sh. Ualikhanov Kokshetau University, 020000, Kokshetau, Kazakhstan
[6] Department of Technology and Standardization, Kazakh University of Technology and Business, 010000, Astana, Kazakhstan
[7] Department of Biomedicine, Karaganda Medical University, 100012, Karaganda, Kazakhstan

*Correspondence: faz8990@mail.ru, ayaulym.mustafa@mail.ru

Analysis of β-CD NMR Spectra

The ^{1}H NMR spectrum of β-CD, obtained in deuterated dimethyl sulfoxide (DMSO), is characterized by six groups of signals in the range of 3.23-3.32; 3.45-3.53; 3.56-3.60; 4.47-4.49; 4.77-4.78; and 5.66-5.73 ppm (Fig. 1). The lowest-field doublet signal in the range of 5.71-5.73 ppm with a splitting of 4 Hz belongs to the proton of the hydroxyl group at the C-2 atom (OH-2). The proton of the OH group of the adjacent atom (OH-3), located in the internal cavity of the CD molecule, also resonates in the downfield region (δ = 5.66 ppm, doublet). The doublet signal in the region of 4.77-4.78 ppm corresponds to the H-1 proton. The location of this proton in a weaker field compared to the protons of other CH groups is due to the influence of the oxygen atom. The hydroxyl group OH-6 resonates, splitting into a triplet centered at 4.48 ppm. In the strong field region (3.56-3.60 ppm), an accumulation of signals of the H-6 a, b methylene group is observed. The high-intensity signal at 3.45 ppm corresponds to the H-3 and H-5 protons of the glucopyranose unit. In the range from 3.23 to 3.32 ppm, the methine protons H-2 and H-4 appear.

The carbon spectrum of β-CD (Fig. 2) consists of five signals from the ^{13}C nuclei of the elementary unit. The upfield signal is due to the C-6 carbon atom (δ = 60.41 ppm). The signals at 72.49, 72.85, and 73.51 ppm are due to the C-5, C-2, and C-3 atoms, respectively. Downfield signals (82.02 and 102.41 ppm) are due to the carbon atoms (C-4 and C-1, respectively), directly linked to the adjacent glucopyranose unit via an oxygen bridge.To obtain more complete and accurate information on homo- (^{1}H-^{1}H) and heteronuclear (^{1}H-^{13}C) correlations, two-dimensional NMR spectra were acquired and interpreted in COSY, HSQC, and HMBC formats. The interactions between the ^{1}H nuclei can be determined using the COSY spectrum shown in Fig. 3. The presence of spin interaction, mediated through 3 bonds, between the proton at the carbon atom of the glucopyranose unit and the proton of the corresponding hydroxyl group is indicated by crosspeaks with coordinates (3.30; 5.79) H2–OH2; (3.63; 4.58) H6–OH6. The correlation with coordinates (3.34; 4.87) corresponds to interaction through 3 bonds between the H-1 and H-2 protons. A similar relationship is observed for the H2–H3, H3–H4, and H4–H5 protons. The interactions of the ^{1}H–^{13}C nuclei, mediated through 1, 2, and 3 bonds, can be traced using the HMBC (Fig. 4) and HSQC spectra (Fig. 5). Heteronuclear interactions through 1 bond (Fig. 5) are characteristic of the nuclei H1–C1 (4.81; 102.46), H2–C2 (3.29; 72.66), H3–C3 (3.61; 73.26), H4–C4 (3.33; 81.82), H5–C5 (3.54; 72.01), H6–C6 (3.62; 60.16). The following interactions are characteristic of the hydroxyl proton at the C2 carbon atom: OH2-C2 and OH2-C3, determined by fragment a (5.74; 72.41), as well as OH2-C1 - the peak with coordinates (5.74; 102.23). It should be noted that the first type of interaction occurs through 2 bonds, and the next two - through 3. The hydroxyl group at the third carbon atom (OH-3) also interacts with neighboring 13C nuclei through two (OH3-C3) and three bonds (OH3-C2; OH3-C4), as evidenced by peaks b (5.68; 72.32) and c (5.68; 81.75). Peak e (4.82; 72.38) corresponds to H1-C2 and H1-C3 interactions, realized through 2 and 3 bonds, respectively. Also, this proton is characterized by a correlation with

the carbon atom C4 d (4.80; 81.39). The hydroxyl group OH-6 is determined by the peaks (4.52; 59.97) and (4.52; 72.13), which correspond to the bonds OH6–C6 (through two bonds) and OH6–C5 (through three bonds). The peak (3.62; 72.53) indicates the interactions of the proton-carbon nuclei H6–C5 (through two bonds). Also, this proton is characterized by correlations with the C-4 atom (3.62; 81.77). Protons H-2 and H-4 interact with carbon atoms C3 and C5, as confirmed by the cross peak (3.33; 73.30). For H-2, a correlation with the C-1 atom at the point (3.33; 102.19) was also determined.

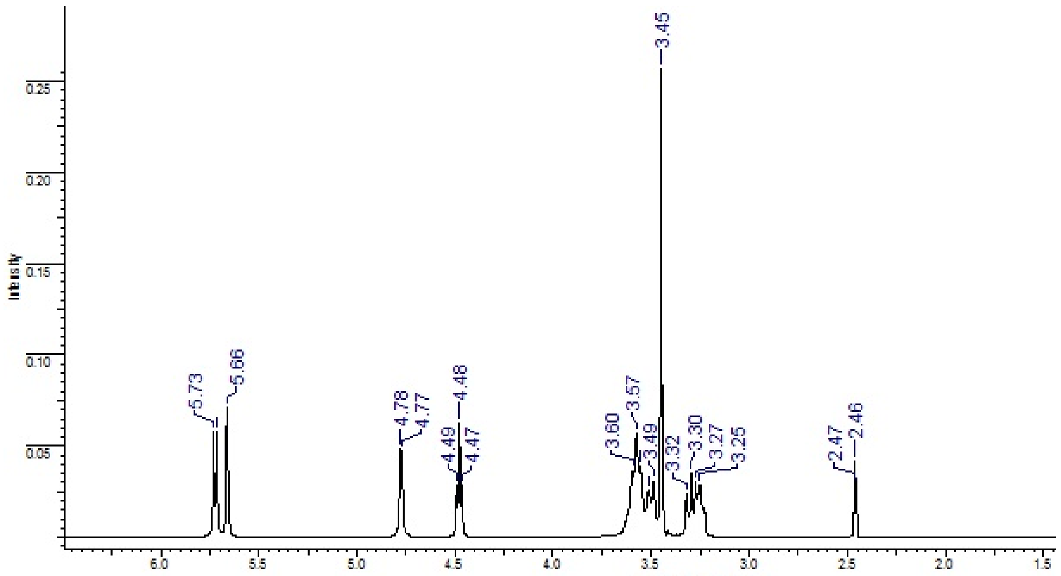

Figure S1. ^{1}H NMR spectra of β-CD (DMSO)

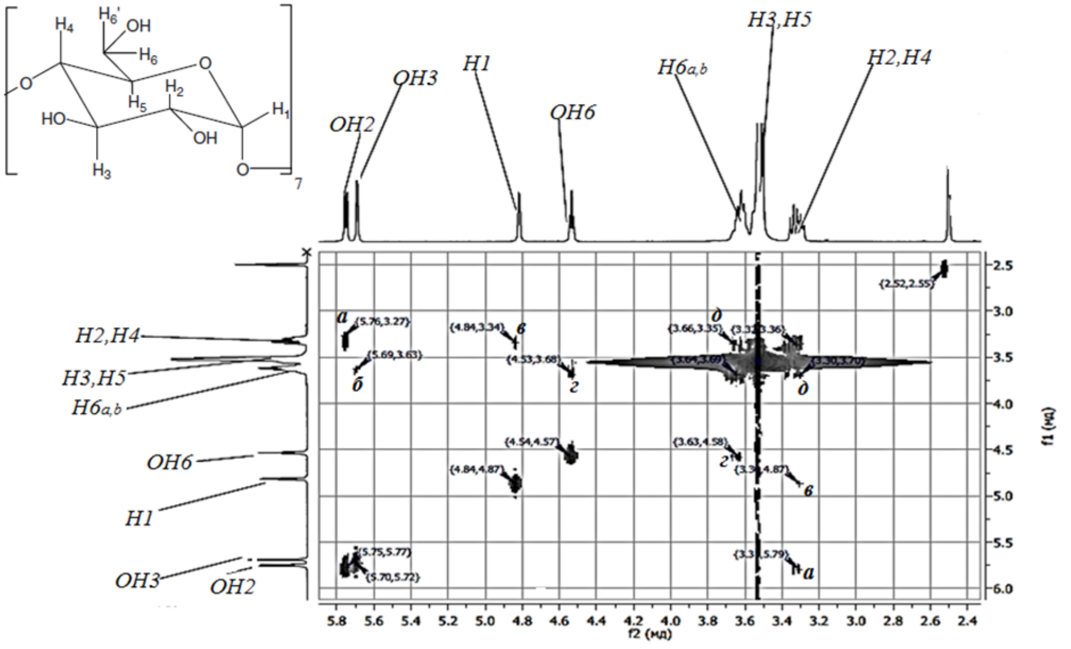

Figure S2. COSY (^{1}H-^{1}H) NMR spectra of β-CD (DMSO)

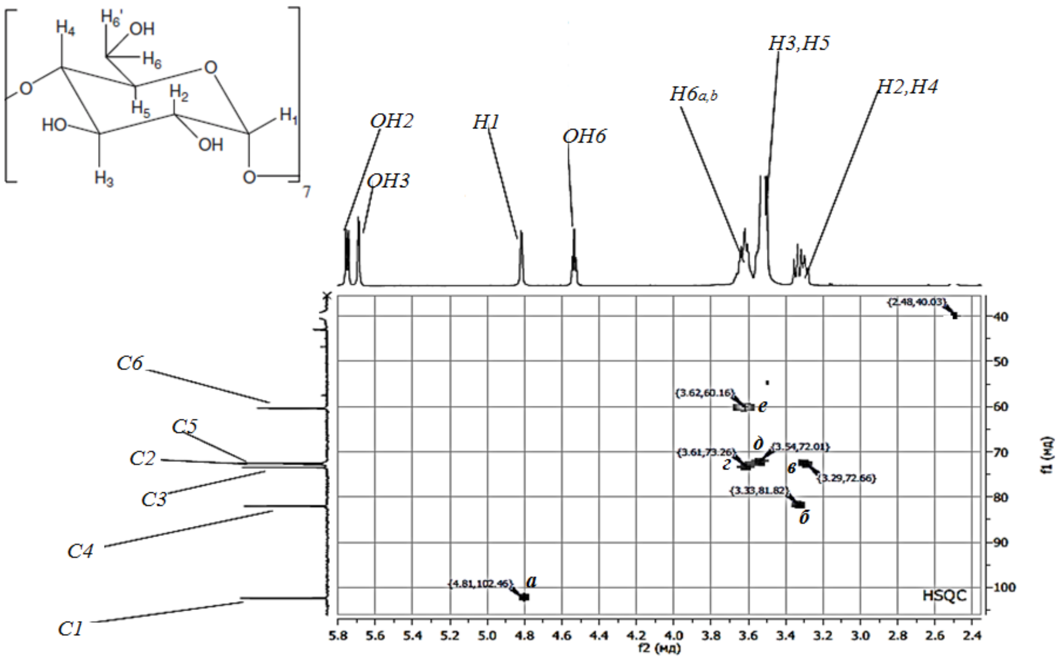

Figure S3. HSQC (^1H-^{13}C) NMR spectra of β-CD (DMSO)

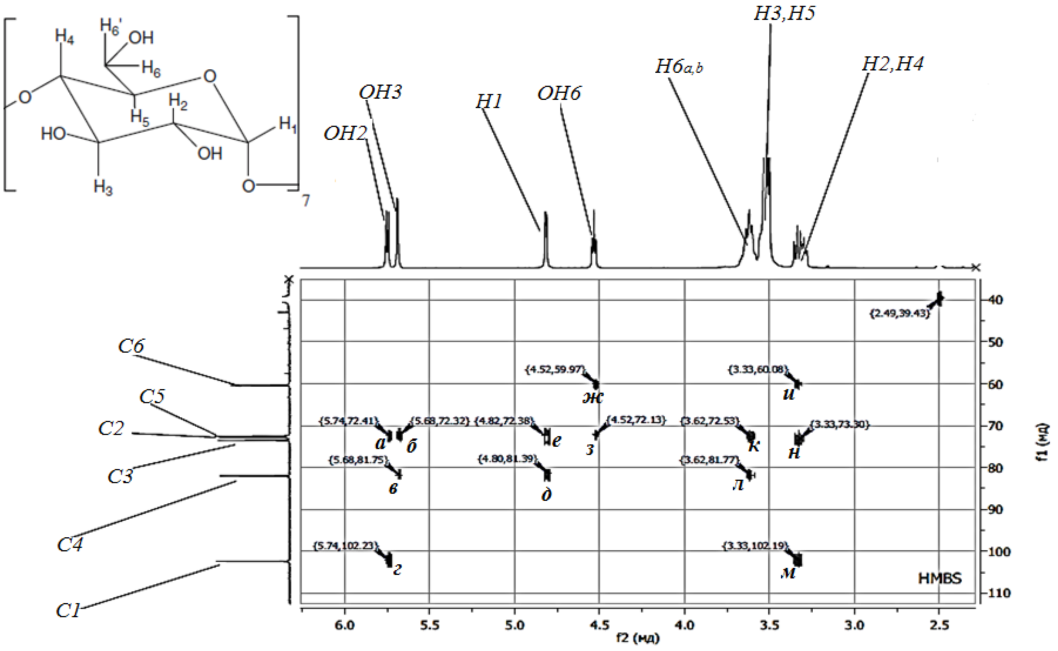

Figure S4. HMBC (^1H-^{13}C) NMR spectra of β-CD (DMSO)

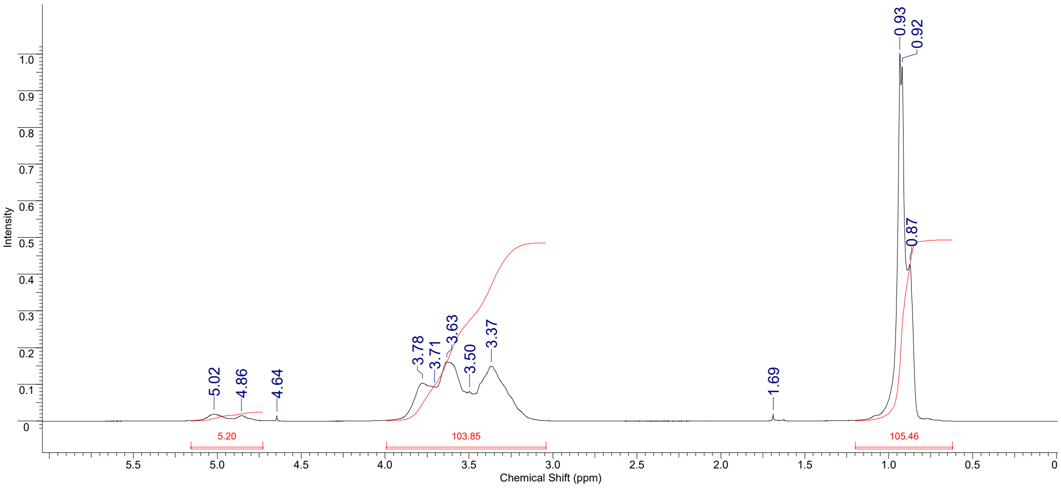

No.	(ppm)	(Hz)	Height
1	0.87	349.4	0.4267
2	0.92	367.2	0.9657
3	0.93	373.2	1.0000
4	1.69	675.0	0.0174
5	3.37	1346.3	0.1493
6	3.50	1397.6	0.0795
7	3.63	1452.1	0.1608
8	3.71	1481.4	0.0925
9	3.78	1510.3	0.1029
10	4.64	1856.9	0.0130
11	4.86	1941.7	0.0139
12	5.02	2006.7	0.0183

No.	(ppm)	Value	Absolute Value
1	[4.73 .. 5.16]	5.200	5.47764e+0
2	[3.04 .. 3.99]	103.846	1.09391e+2
3	[0.62 .. 1.20]	105.463	1.11094e+2

(a)

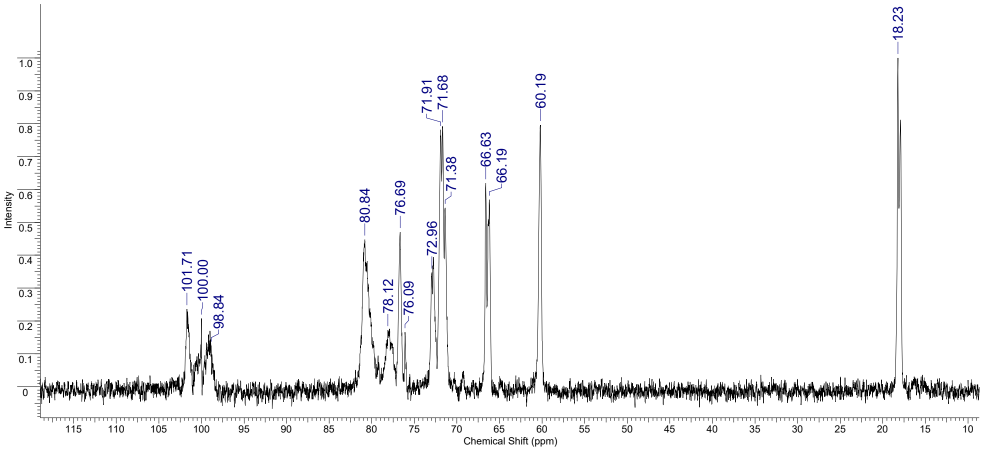

No.	(ppm)	(Hz)	Height
1	18.23	1832.7	1.0000
2	60.19	6050.9	0.7959
3	66.19	6653.8	0.5692
4	66.63	6697.9	0.6187
5	71.38	7175.2	0.5437
6	71.68	7205.9	0.7920
7	71.91	7228.9	0.7810
8	72.96	7334.3	0.3479

No.	(ppm)	(Hz)	Height
9	76.09	7648.7	0.1657
10	76.69	7709.1	0.4712
11	78.12	7852.8	0.1723
12	80.84	8126.0	0.4477
13	98.84	9935.6	0.1255
14	100.00	10052.5	0.2080
15	101.71	10224.1	0.2363

(b)

Figure S5. ^{1}H(a), ^{13}C(b) NMR spectra of β-CD-AgNPs (D_2O)

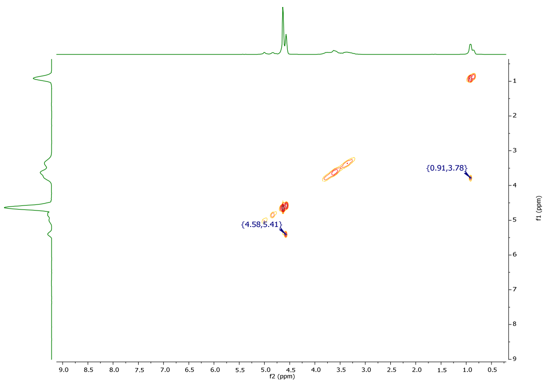

(a)

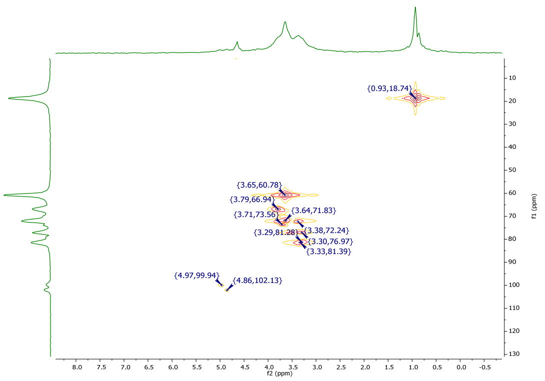

(b)

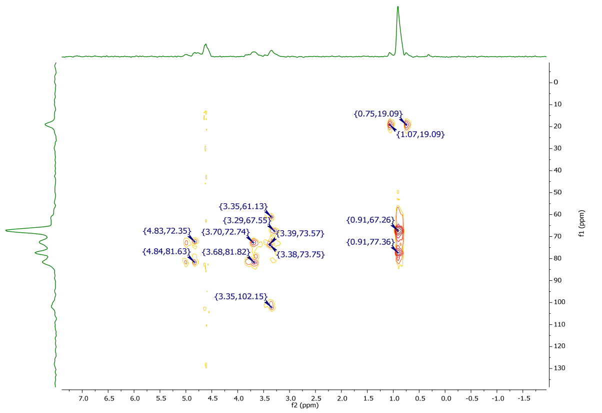

(c)

Figure S6 (a-c). COSY (a), HMQC (b), HMBC (c) spectra of β-CD-AgNPs, (D_2O)

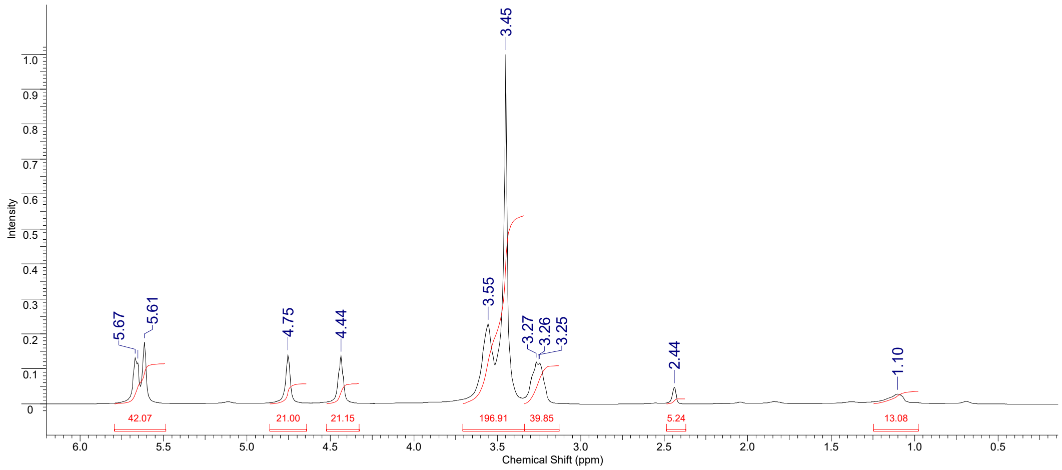

No.	(ppm)	(Hz)	Height
1	1.10	440.5	0.0276
2	2.44	974.9	0.0470
3	3.25	1297.3	0.1167
4	3.26	1301.9	0.1143
5	3.27	1306.0	0.1205
6	3.45	1378.9	1.0000
7	3.55	1421.0	0.2281
8	4.44	1773.6	0.1371
9	4.75	1900.5	0.1409
10	5.61	2244.4	0.1751
11	5.65	2260.4	0.1172
12	5.67	2266.4	0.1314

No.	(ppm)	Value	Absolute Value
1	[2.37 .. 2.49]	5.235	4.18872e+0
2	[0.98 .. 1.25]	13.077	1.04633e+1
3	[4.64 .. 4.86]	21.001	1.68027e+1
4	[4.33 .. 4.52]	21.150	1.69226e+1
5	[3.13 .. 3.34]	39.847	3.18815e+1
6	[5.49 .. 5.79]	42.072	3.36617e+1
7	[3.34 .. 3.71]	196.908	1.57547e+2

(a)

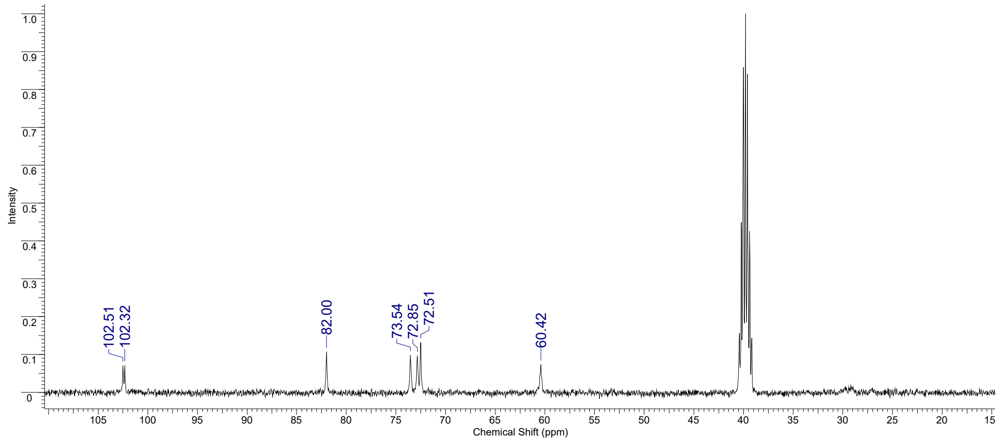

No.	(ppm)	(Hz)	Height
1	60.42	6073.9	0.0734
2	72.51	7289.3	0.1322
3	72.85	7322.8	0.0971
4	73.54	7392.8	0.0981
5	82.00	8242.9	0.1072
6	102.32	10285.4	0.0713
7	102.51	10304.6	0.0705

(b)

Figure S7 (a, b). ^{1}H (a) and ^{13}C(b) NMR spectra of VD_3 (DMSO)

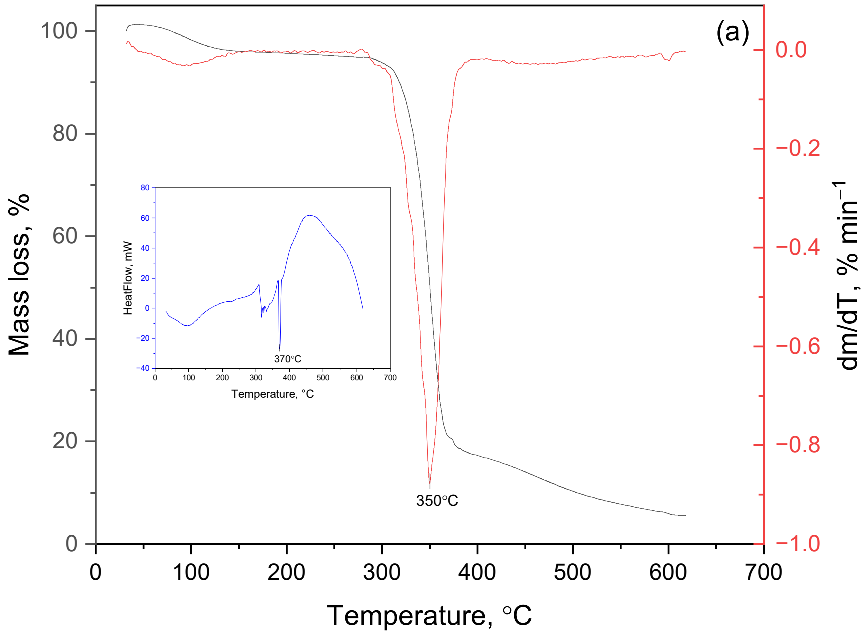

(a)

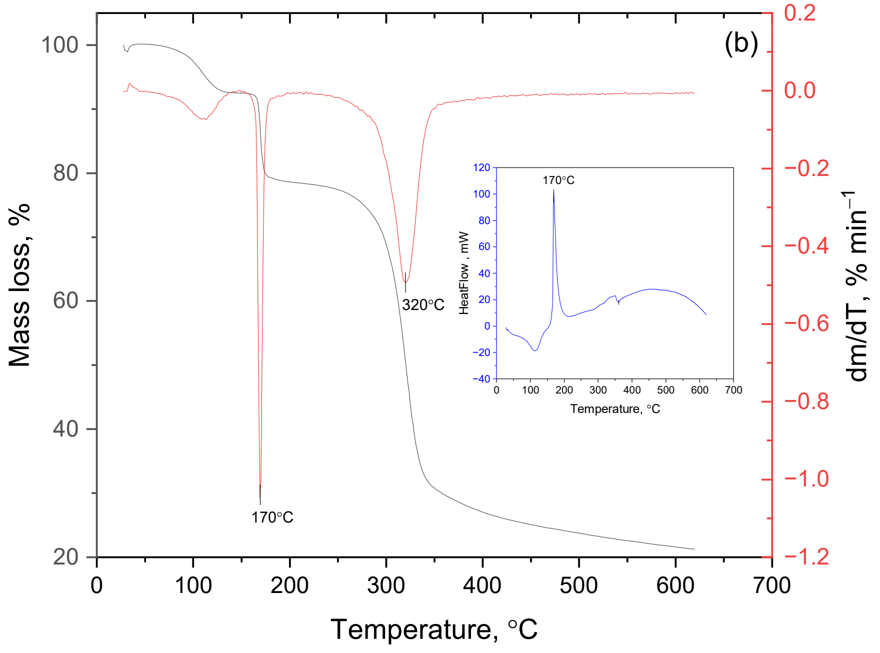

(b)

Figure S8 (a,b). Graphical dependences of mass change on temperature: (a) β-CD–Ag (15:1) and (b) VD_3–β-CD–Ag (15:1:1)

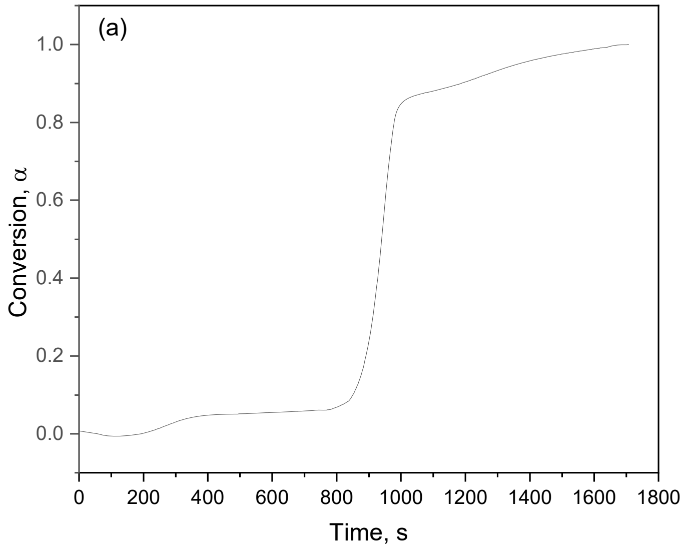

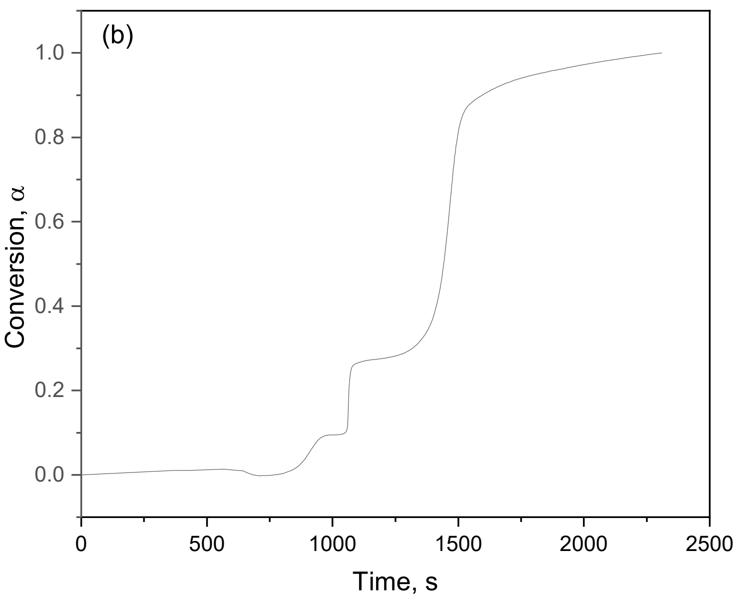

Figure S9. Kinetic curves of the dependence of the degree of transformation on time during thermal decomposition of β-CD–Ag (15:1) (a) and VD_3–β-CD–Ag (15:1:1) (b)
